# Predicting all-cause 90-day hospital readmission for dental patients using machine learning methods

**DOI:** 10.1038/s41405-021-00057-6

**Published:** 2021-01-22

**Authors:** Wei Li, Martin S. Lipsky, Eric S. Hon, Weicong Su, Sharon Su, Yao He, Richard Holubkov, Xiaoming Sheng, Man Hung

**Affiliations:** 1 University of Utah School of Medicine, Salt Lake City, UT USA; 2 Roseman University of Health Sciences College of Dental Medicine, South Jordan, UT USA; 3 University of Chicago Department of Economics, Chicago, IL USA; 4 University of Utah Department of Mathematics, Salt Lake City, UT USA; 5 University of Utah Alzheimer’s Center, Salt Lake City, UT USA; 6 University of Utah College of Nursing, Salt Lake City, UT USA; 7 George E. Wahlen Department of Veterans Affairs Medical Center, Salt Lake City, UT USA; 8 Towson University Department of Occupational Therapy & Occupational Sciences, Towson, MD USA; 9 Utah Center for Clinical & Translational Science, Salt Lake City, UT USA

**Keywords:** Dental conditions, Oral diseases

## Abstract

**Introduction:**

Hospital readmission rates are an indicator of the health care quality provided by hospitals. Applying machine learning (ML) to a hospital readmission database offers the potential to identify patients at the highest risk for readmission. However, few studies applied ML methods to predict hospital readmission. This study sought to assess ML as a tool to develop prediction models for all-cause 90-day hospital readmission for dental patients.

**Methods:**

Using the 2013 Nationwide Readmissions Database (NRD), the study identified 9260 cases for all-cause 90-day index admission for dental patients. Five ML classification algorithms including decision tree, logistic regression, support vector machine, k-nearest neighbors, and artificial neural network (ANN) were implemented to build predictive models. The model performance was estimated and compared by using area under the receiver operating characteristic curve (AUC), and accuracy, sensitivity, specificity, and precision.

**Results:**

Hospital readmission within 90 days occurred in 1746 cases (18.9%). Total charges, number of diagnosis, age, number of chronic conditions, length of hospital stays, number of procedures, primary expected payer, and severity of illness emerged as the top eight important features in all-cause 90-day hospital readmission. All models had similar performance with ANN (AUC = 0.743) slightly outperforming the rest.

**Conclusion:**

This study demonstrates a potential annual saving of over $500 million if all of the 90-day readmission cases could be prevented for 21 states represented in the NRD. Among the methods used, the prediction model built by ANN exhibited the best performance. Further testing using ANN and other methods can help to assess important readmission risk factors and to target interventions to those at the greatest risk.

## Introduction

Hospital readmission within 90-day of an index hospitalization may result from actions or inactions taken during the initial hospital stay.^1^ Since Centers for Medicare & Medicaid began publishing readmission data in 2009, this metric quickly became viewed as an indicator of health care quality and cost provided by hospitals.^2,3^ Accordingly, reducing unnecessary readmissions became a key concern for healthcare providers and payors.^2,4^

Accurate models to predict hospital readmission offer the potential to identify those patients at highest risk and for addressing factors associated with avoidable readmissions. Nevertheless, success in predicting and reducing readmission rates remains a work in progress. While readmission rates from 2010 to 2016 decreased 7% for Medicare patients, Medicaid and privately insured patients did not experience a similar decline and readmission rates for uninsured patients^5^ actually increased by 14%.^6^

The recent availability of large databases and enhanced computing power^7,8^ suggests that the application of artificial intelligence (AI) and machine learning (ML) methods offer the potential to accurately predict those patients at highest risk for readmission.^5,9–13^ Compared to traditional statistical approaches, ML builds complex algorithms based on data of either parametric or nonparametric nature. The learning process can identify patterns in data to forecast future events. As an example, Mahajana et al. applied two ensemble schemes of ML models to predict the risk of readmission for heart failure using Electronic Health Records (EHR).^14^ Similarly, Bayati et al. constructed a predictive model for congestive heart failure readmissions with EHR data. They applied the least absolute shrinkage and selection operator technique to select the most predictive variables and employed the LACE index (length of stay, acuity of admission, Charlson comorbidity index, and number of emergency department visits in preceding 6 months) to build their prediction models. He et al. created an algorithm to predict 30-day readmission using an all-inclusive Johns Hopkins Hospital cohort and testing it on the entire Bayview Medical Center cohort. Futoma et al. compared several existing health related prediction models and suggested a simple framework to improve medical decision making by using random forests (RF) and deep learning methods.^13^

Most published research regarding readmission focused on medical and surgical conditions. However, while these are critically important areas, oral health is often an overlooked component of overall health care. In the US, more than $124 billion is spent on oral health related conditions annually and Healthy People 2020 lists improving oral health as one of its key objectives. As a result, there is an increasing interest in applying ML techniques to build prediction models related to dental care area.^14^ Despite the importance of oral health few studies address hospital readmission linked to dental health, and no published study explored the application of ML methods to develop a predictive model related to oral healthcare associated hospital readmission.

This study aimed to be the first study to use ML methods to develop a prediction model for all-cause 90-day hospital readmission for dental patients. Many studies used the 30-day readmission metric but few studies examine the 90-day metric even though hospital readmissions within the first 90-days were quite common. Previously studies found that the 30-day readmission rate for dental-related conditions was about 11%,^15^ however this study represents the first to examine the important 90-day dental hospital readmission metrics using the most relevant variables available in the National Readmissions Database (NRD), including demographic, socioeconomic and clinical factors. An additional aim is to evaluate and compare the performance of several ML methods to predict dental hospital readmission. Developing this prediction model should provide a useful tool for reducing hospital readmission for dental-related patients.

## Methods

### Data source and study group

The NRD is a part of the Healthcare Cost and Utilization Project (HCUP) and is designed to provide nationally representative information on hospital readmissions for all types of payers and the uninsured. It also includes reasons for returning to the hospital for care and the estimation of readmission rates.^3,16^ This study used the 2013 NRD, which contains data for 14,325,172 hospital discharges. The project integrated four data files (Core file, severity file, diagnostic and procedure code file, hospital characteristics file) from NRD into a unified dataset. The ICD-9-CM dental diagnostic codes (starting with 52) and procedure codes (starting with 23, 24 and 27), and some selected codes (starting with 96, 97 and 99) were used to identify dental inpatients for inclusion into this study. This study excluded patients who died during a hospital stay, hospital stays <1 day, and those discharged after September (for 90-day readmission). Index events were identified by using inclusive and exclusive criteria stated above.

After applying the inclusion and exclusion criteria, 9677 cases identified for all cause 90-day index admissions. These 90-day dental hospital admission cases represented ~0.07% of all hospital admission cases. There were 236 variables in these initial data sets. The ID variables that were unlikely to be related to hospital readmission, variables highly correlating with other variables in the dataset, variable weights, as well as variables that were used to select specific patients, were eliminated. A total of 56 variables remained in the dataset including the outcome variable (Appendix A). In order to prepare for further variable selection and preliminary analyses, the application of listwise deletion eliminated a small portion of cases with missing data. A final total of 9260 cases remained in the 90-day hospital readmission dataset.

### Outcome measures

The primary outcome measures were readmissions within 90 days following discharge of an index hospitalization for patients admitted with dental-related conditions. In total, the study identified 1746 cases (18.9%) for all cause 90-day hospital readmissions for dental patients over the study period.

### Candidate predictors

Based on literature review and expert clinical consultation, the study defined three broadly based categories of 55 applicable variables:^5,10^ demographic category (e.g., age and gender); socioeconomic category (e.g., median household income, primary payer, patient location, total charges, etc.); and clinical category (e.g., length of hospital stay, hospital urban-rural designation, emergency department service indicator, number of chronic conditions, number of diagnosis, number of procedures, AHRQ comorbidity measures, etc.).

Traditional statistical methods were used to further filter these variables. Continuous variables and categorical variables were tested to examine whether there were significant differences at α = 0.05 between the readmission group and non-readmission group for each variable based on a combination of variable type and sample sizes. For categorical variables, the analyses used Fisher’s exact test for total sample sizes <30 or expected cell counts of <5, or alternatively a simulated Chi-squared test was applied when the dataset was too large for Fisher’s exact test; otherwise the analyses used a Chi-squared test. For continuous variables, a *t*-test was used if the variable was normally distributed; otherwise a Wilcoxon rank sum test was used. In total, the selection process identified 47 variables with statistically significant relationships to the outcome variable Readmission (*p* value < 0.05) into the 90-day preliminary models (Appendices B and C). Statistical analyses were done using the R software.

### Machine learning methods

In formulating the ML model, the raw dataset was split into two mutually exclusive sets, a training set (70%) and a testing set (30%). The training set was used to generate the prediction classifier and the testing set was used to estimate the models’ performances.

The data revealed an imbalanced binary outcome for the 90-day (18.9% having readmission) readmission data sets. An imbalanced training set provides less information on the minority class and can bias prediction and cause inaccuracies of the ML models. Minority class refers to the category of the outcome variable that has fewer cases. For example, the outcome variable in this study had two categories:^1^ Readmitted, and^2^ Not Readmitted. The “Readmitted” category contained 18.9% of cases, which was less than the “Not readmitted” category of 81.1%. Thus, the “Readmitted” category was the minority class. In order to adjust for the imbalanced dataset, data resampling techniques were applied to balance the data. The balanced training sets and the logistic regression (LR) method were used to build the prediction models, and the performance of these prediction models was then tested on the test data, and evaluated by using the area under the Receiver Operating Characteristic (ROC) curve (AUC).

Data standardization and normalization are required for some ML methods (e.g., Support Vector Machine (SVM)), but not for the tree-based decision algorithms like decision trees (DT) or RF. This study applied *Z*-score standardization for continuous predictors and dummy coding for the categorical variables. The standardized train dataset was utilized in the LR, SVM, k-Nearest Neighbor (k-NN) and Artificial Neural Network (ANN) ML methods.

RF, a tree-based ML algorithm, also known as an ensemble learning method, can be applied to both classification and regression tasks. Bagging (or bootstrap aggregating) technique was used in RF to build independent identical distributed trees and to grow deep trees, and to reduce the variance of an estimated prediction function.^17^ During the construction of RF, the out-of-bag (OOB) error rate was calculated by averaging the prediction error of each unselected training sample through the bootstrap sampling for training. Aggregation of predictions can help reduce both bias and variance.

As a supervised learning technique (the presence of the outcome variable guides the learning process,^17^ one popular application of RF is the important feature selection. Feature selection helps to reduce the dimensions without much loss of the information, leads to a decrease in training time and model complexity and prevents the data overfitting. This study used the *randomForest* package in R to select the important features for the ML models. As an ensemble of individual DT, RF also uses the Gini Impurity to evaluate the importance of a variable on predicting the outcome across all of the individual trees in the forest.

The study applied the training set and five supervised ML algorithms (LR, DT, SVM, k-NN, and ANN) to train and build prediction classifiers. The k-fold validation was then employed to estimate the ML model on unseen data (test data) and model performances on the testing set evaluated by using accuracy, sensitivity, specificity, precision, and the AUC.

## Results

**Fig. 1:**
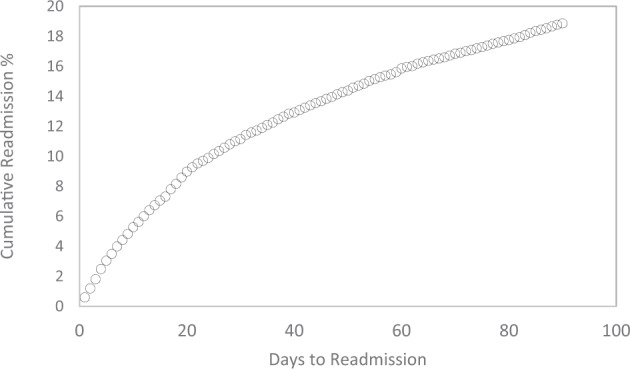
Cumulative readmission rate by days to readmission after hospital discharge. Circle symbols indicate the cumulative readmission percentage (%) from 0 to 90 days after hospital discharge.

**Fig. 2:**
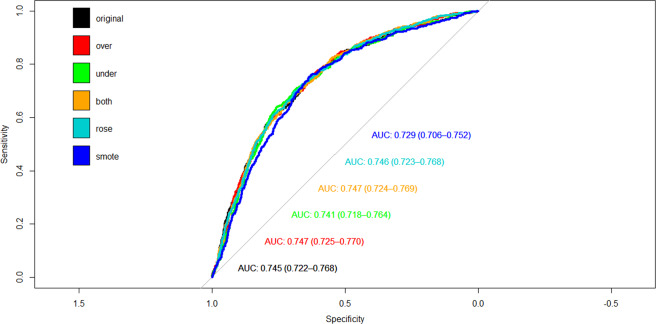
ROC and AUC of logistic regression models by using balanced dataset and the original dataset. The oversampling (Over), undersampling (Under), bothsampling (Both), random oversampling examples (ROSE), and synthetic minority over-sampling technique (SMOTE) were applied to balance the dataset.

**Fig. 3:**
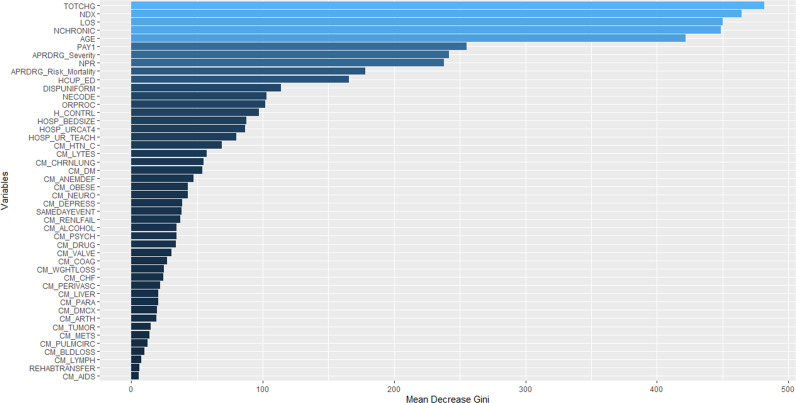
Important feature selection by using random forest. Blue bars show the mean decreased Gini index for 46 predictor candidates.

Figure 1 shows the relationship between the cumulative readmission percentage (%) and the number of days after hospital discharge. The cumulative readmission reaches 18.9% at 90 days after discharge. Figure 2 summarizes the ROC curves and associated AUC of LR models trained by the balanced data sets and 46 predictors, and displays oversampling balanced data with the highest AUC. Using their contribution to the mean decreased Gini index, the top eight most important features selected by random forest included total charges, number of diagnosis on this record, age in years at admission, number of chronic conditions, length of hospital stay, number of procedures on this record, primary expected payer, and severity of illness subclass (Fig. 3). These selected features were used to construct the preliminary model for ML methods. The RF classifier generated 500 different trees and sampled 6 variables at each split, and the OOB estimated error rate was 4.63%. According to the plots of error by trees, there were apparent flatlines after 200 built trees in both forests. By using 200 trees instead of 500 trees, the OOB error rate increased only slightly to 4.9%, while the important features did not change.

Among the 9260 patients’ records, 18.9% (*n* = 1746) had hospital readmissions. Table 1 presents a descriptive summary of patient characteristics. The sample consisted of 55.8% male (*n* = 5167). The mean age at admission was 42.1 years (SD = 20.9; median = 42; range = 0–90). The mean length of hospital stay was 7.9 days (SD = 14.7; median = 3; range = 1–6). The average total medical charges were $76,009 with a median $34,063. The mean number of diagnoses for individual was 9.6 (SD = 6.3; median = 8; range = 5–14) and number of procedures 3.8 (SD = 3.1; median = 3; range = 2–5). The mean number of chronic conditions was 3.7 (SD = 3.3; median = 3; range = 1–6). Insurance coverage included 22.7% Medicare (*n* = 2099), 29% Medicaid (*n* = 2688), 24.4% private insurance (*n* = 2264), 15.6% with self-pay (*n* = 1448) and 8.2% with either no charge or other payer (*n* = 761). For illness severity of illness, 39.2% (*n* = 3626) experienced minor loss of function and 33.8% (*n* = 3126) moderate loss of function. A median household income in the lowest quartile comprised about one third of the patients (33.7%, *n* = 3124) and compared to 18% (*n* = 1666) in the highest quartile. In general, the 90-day readmitted patients were older, had a longer length of hospital stay initially, had more diagnoses and procedures on record and had greater number of chronic conditions (Table 1). There was a total of 1746 patients with 90-day readmissions, but if none of these 1746 patients required hospital readmission, there would be a cost saving of $103,272,408 in hospital charges.

**Table 1:** Descriptive summary of the 90-day dental hospital readmission data.

Variable	Total Cohort (*N* = 9260)	Readmitted (*N* = 1746)	Not Readmitted (*N* = 7514)	*P* value
*Demographic factors*				
Age in years at admission: Mean (SD)	42.1 (20.9)	49.8 (20.1)	40.4 (20.6)	<0.001^a^
Median (IQR)	42.0 (26.0, 57.0)			
Range	(0.0, 90.0)			
Gender: Male	5167 (55.8%)	990 (56.7%)	4177 (55.6%)	0.40^b^
Female	4093 (44.2%)	756 (43.3%)	3337 (44.4%)	–
*Socioeconomic factors*				
Total charges: Mean (SD)	76,009.7 (142,509.6)	124,005.3 (216,769.3)	64,857.2 (115996.4)	<0.001^a^
Median (IQR)	34,063.0 (18,732.8, 72,658.2)			
Range	(473.0, 4580,711.0)			
Primary expected payer: Medicare	2099 (22.7%)	645 (36.9%)	1,454 (19.4%)	<0.001^b^
Medicaid	2688 (29%)	555 (31.8%)	2133 (28.4%)	–
Private insurance	2264 (24.4%)	310 (17.8%)	1954 (26%)	–
Self-pay	1448 (15.6%)	136 (7.8%)	1312 (17.5%)	–
No charge	166 (1.8%)	21 (1.2%)	145 (1.9%)	
Other	595 (6.4%)	79 (4.5%)	516 (6.9%)	–
Median household income: $1–$37,999	3124 (33.7%)	581 (33.3%)	2543 (33.8%)	0.60^b^
$38,000–$47,999	2391 (25.8%)	436 (25%)	1955 (26%)	–
$48,000–$63,999	2079 (22.5%)	410 (23.5%)	1669 (22.2%)	–
$64,000 or more	1666 (18%)	319 (18.3%)	1347 (17.9%)	–
*Clinical factors*				
Length of stay: Mean (SD)	7.9 (14.7)	12.8 (16.9)	6.7 (13.9)	<0.001^a^
Median (IQR)	4.0 (2.0, 8.0)			
Range	(1.0, 340.0)			
Number of diagnosis on this record: Mean (SD)	9.6 (6.3)	14.0 (9.0, 18.0)	7.0 (4.0, 12.0)	<0.001^c^
Median (IQR)	8.0 (5.0, 14.0)			
Range	(1.0, 25.0)			
Number of chronic conditions: Mean (SD)	3.7 (3.3)	6.0 (3.0, 8.0)	2.0 (1.0, 5.0)	<0.001^c^
Median (IQR)	3.0 (1.0, 6.0)			
Range	(0.0, 17.0)			
Severity of Illness Subclass: Minor loss of function	3626 (39.2%)	270 (15.5%)	3,356 (44.7%)	<0.001^b^
Moderate loss of function	3126 (33.8%)	566 (32.4%)	2,560 (34.1%)	–
Major loss of function	1797 (19.4%)	633 (36.3%)	1,164 (15.5%)	–
Extreme loss of function	711 (7.7%)	277 (15.9%)	434 (5.8%)	–
Number of procedures on this record: Mean (SD)	3.8 (3.1)	4.0 (2.0, 6.0)	3.0 (2.0, 4.0)	<0.001^c^
Median (IQR)	3.0 (2.0, 5.0)			
Range	(1.0, 15.0)			

^a^Chi-squared test.

^b^*T*-test.

^c^Wilcoxon rank sum test.

**Fig. 4:**
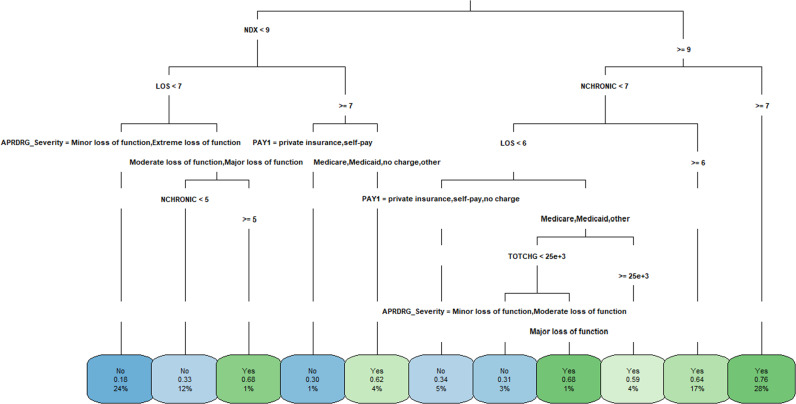
Decision Trees for 90-day readmission data sets. A feature with higher entropy is located closer to the root (top), and a branch with zero entropy is converted to a leaf node (bottom). Each leaf node displays the probability of the “Yes” or “No” class at that node and the percentage of total observations used at that node.

**Fig. 5:**
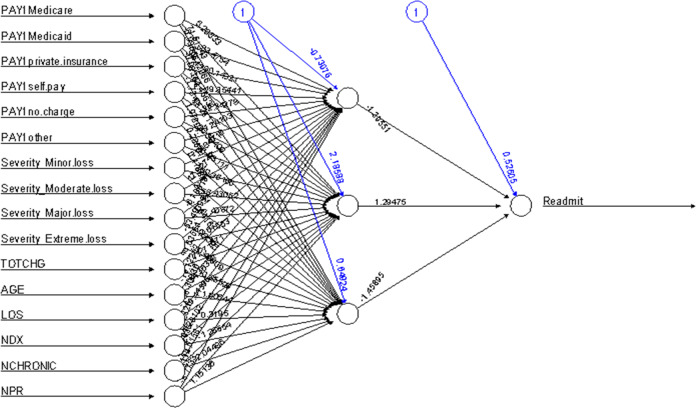
ANN for 90-day readmission data sets. ANN structure: 16-3-1. The optimal weights of input variables and intercepts are shown as black and blue numbers above the lines separately.

**Fig. 6:**
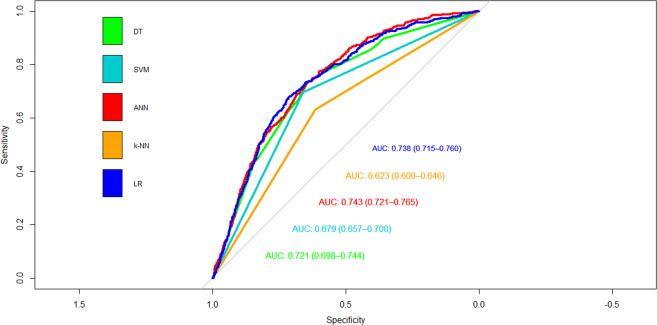
AUC of ML methods for the 90-day readmission. Five supervised machine learning algorithms: Decision Tree (DT), Support Vector Machine (SVM), Artificial Neural Network (ANN), k-Nearest Neighbor (k-NN), and Logistic Regression (LR) are applied to build prediction models.

Figure 4 illustrates the decision tree results and Fig. 5 demonstrates the ANN plots for the 90-day readmission data. Figure 6 depicts the ROC curves and the area under ROC curves (AUC) for each ML method. Table 2 summarizes the performance of each ML method, including sensitivity, specificity, accuracy, precision and AUC. For the 90-day readmission data, the ANN performed the best (AUC = 0.743), followed by LR (AUC = 0.738), DT (AUC = 0.721), SVM (AUC = 0.679) and k-NN(AUC = 0.623). DT got the highest sensitivity (0.734), followed by ANN (0.719). SVM and ANN had similar accuracy (0.667 and 0.665).

**Table 2:** Performance metrics of ML models for the 90-day readmission data.

Classifier	Accuracy	Sensitivity	Specificity	Precision	AUC
Decision tree	0.659	0.734	0.642	0.316	0.721
Support vector machine	0.667	0.696	0.661	0.317	0.679
k-Nearest neighbors	0.610	0.645	0.602	0.268	0.623
Artificial neural network	0.665	0.719	0.653	0.319	0.743
Logistic regression	0.688	0.698	0.686	0.334	0.738

## Discussion

A 2009 landmark article by Jencks et. al found that about 1 in 3 Medicare patients discharged from the hospital were readmitted within 90 days.^18^ With the prevalence of hospital readmission and the associated costs, hospital readmission became the focus of the Center of Medicare and Medicaid Services, initially concentrating on heart disease and pneumonia but more recently expanding to other medical conditions. Predictive models can help characterize risk factors and by identifying those most at risk can help develop targeted interventions. It also provides metrics to help assess quality and cost.

Despite the importance of dental health to one’s general health, few studies examine dental-related hospitalization^19^ and there is a paucity of research related to predictive models and AI tools for hospital readmission following dent-related procedures and conditions. This is the first study to use ML methods and AI to explore dental readmissions in the United States. Our results indicate that about 18.9% of dental patients were readmitted within 90 days, a finding that helps establish a basis to assess intervention. Those insured by Medicare and Medicaid experienced higher hospital readmission rates than those with other insurances. While the exact percentage of preventable hospital readmissions is unclear, our study illustrates that the annual cost savings for dental readmissions just with the 21 states represented in the NRD could exceed $500 million dollars if all readmissions could be prevented. A more conservative estimate that 1 in 4 rehospitalizations might be prevented and would still result in savings of over $100 million.^4^ Furthermore, this study supports the current literature that age, length of initial hospital stay, number of diagnoses and procedures, and number of chronic conditions correlated with increased 90 day readmission risk.

Despite many attempts to develop accurate prediction models for hospital readmissions, to date most models demonstrated either mixed results or performed poorly.^12^ Notwithstanding the usefulness of prediction models in clinical settings, the failure to find good models may be due to the broad list of factors and complex interactions involved in why someone is readmitted. Compared to the traditional analytic methods of standard predictive models, this novel study applied four ML models utilizing a selection of eight important features to predict 90-day readmissions for dental patients. Socioeconomic factors were among the 46 significant predictors of the readmissions, but were not ranked as the top eight. The highest AUC was reached at around 0.743 using prediction models built by ANN. The decision tree also had a good AUC of about 0.721. The best results came from using ANN or DT classifiers to build the prediction model for the NRD hospital readmission. These findings suggest that applying these tools to dental hospitalizations might identify which specific individuals to target for intervention.

### Limitations

The population, setting, and condition are factors that affect the best method to build a model. Additional ML analyses and interactions such as testing different feature selection sets, using different classifiers and multiple feature combinations could refine future models and improve prediction performance. However, this could require many hours repeated testing and enhanced computing power to achieve. Future research can help continue to search for and refine models and algorithms for better results. Another limitation was that some numeric feature distributions were highly right skewed and it could be better to use log transformed data. However, interpreting the results of the transformed data is often difficult. In addition, although this study examined risk factors, it failed to explore the specific dental conditions related to the readmissions. The NRD database represents a single year of data, so trends and consistency of data could not be extrapolated. Furthermore, the NRD database does not include readmission data from other states outside the included 21 states from the State Inpatient Database.

## Conclusion

The 90-day readmission rate for dental-related hospitalizations is 18.9%. Readmission was associated with older age, severity of illness, number of diagnoses, procedures, and chronic conditions, length of hospital stay, and having Medicare or Medicaid insurance. The finding that dental readmission was associated with the number of diagnoses and chronic conditions provides empirical evidence that dental health is indeed linked to overall health and general health. The prediction models built by ANN reached the highest AUC around 0.743 among the four ML methods. The decision tree also had a good AUC of about 0.721 with an easily interpreted tree. Using the ANN or DT classifiers to build the prediction model for the NRD hospital readmission data is recommended.

## Supplementary information

Appendix A

Appendix B

Appendix C

## Supplementary information

**Supplementary information** The online version contains supplementary material available at 10.1038/s41405-021-00057-6.
