# Supplementary material for: Predicting all-cause 90-day hospital readmission for dental patients using machine learning methods
Source: BDJ Open. 2021 Jan 22;7:1. doi: 10.1038/s41405-021-00057-6 (PMC7822935; doi:10.1038/s41405-021-00057-6)
Supplement: Supplementary file 1 — Appendix A [file 41405_2021_57_MOESM1_ESM.docx]

**Appendix A: Code book for 56 candidate variables in the 2013 NRD data set**

| **Variable Name** | **Variable Label** | **Value Label** |
| --- | --- | --- |
| LOS | Length of stay (cleaned) |  |
| NRD_DaysToEvent | Timing variable used to identify days between admissions |  |
| Readmit | Indicator of hospital readmission | (0) no hospital readmission, (1) hospital readmission |
| HOSP_BEDSIZE | Bed size of hospital | (1) small, (2) medium, (3) large. |
| H_CONTRL | Control/ownership of hospital | (1) government, nonfederal [public]; (2) private, not-for-profit [voluntary]; (3) private, investor-owned [proprietary] |
| HOSP_URCAT4 | Hospital urban-rural designation | (1) large metropolitan areas with at least 1 million residents, (2) small metropolitan areas with less than 1 million residents, (3) micropolitan areas, (4) not metropolitan or micropolitan, (8) metropolitan, collapsed category of large and small metropolitan, (9) non-metropolitan, collapsed category of micropolitan and rural |
| HOSP_UR_TEACH | Teaching status of urban hospitals | (0) metropolitan non-teaching, (1) metropolitan teaching, (2) non-metropolitan |
| AGE | Age in years at admission | Age in years coded 0-90 years; any age greater than 90 was set to 90. |
| AWEEKEND | Admission day is a weekend | (0) admission on Monday–Friday, (1) admission on Saturday–Sunday |
| DISPUNIFORM | Disposition of patient (uniform) | (1) routine, (2) transfer to short term hospital, (5) other transfers, including skilled nursing facility, intermediate care, and another type of facility, (6) home health care, (7) against medical advice, (20) died in hospital, (99) discharged alive, destination unknown |
| DQTR | Discharge quarter | (1) Jan–Mar, (2) Apr–Jun, (3) Jul–Sep, (4) Oct–Dec |
| FEMALE | Indicator of sex | (0) male, (1) female |
| HCUP_ED | HCUP Emergency Department service indicator | (0) record does not meet any HCUP ED criteria, (1) ED revenue code was on SID record, (2) ED charge reported on SID record, (3) ED CPT procedure code on SID record, (4) other indication of ED services |
| NCHRONIC | Number of chronic conditions |  |
| NDX | Number of diagnoses on this record |  |
| NECODE | Number of E codes on this record |  |
| NPR | Number of procedures on this record |  |
| ORPROC | Major operating room procedure indicator | (1) major operating room procedure reported on discharge record, (0) no major operating room procedure reported on discharge record |
| PAY1 | Primary expected payer (uniform) | (1) Medicare, (2) Medicaid, (3) private insurance, (4) self-pay, (5) no charge, (6) other |
| PL_NCHS | Patient Location: NCHS Urban-Rural Code | (1) "Central" counties of metro areas of >=1 million population,(2) "Fringe" counties of metro areas of >=1 million population,(3) Counties in metro areas of 250,000–999,999 population,(4) Counties in metro areas of 50,000–249,999 population,(5) Micropolitan counties,(6) Not metropolitan or micropolitan counties |
| REHABTRANSFER | A combined record involving rehab transfer | (1) yes, (0) no |
| RESIDENT | Patient State is the same as Hospital State | (1) resident, (0) nonresident |
| SAMEDAYEVENT | Transfer flag indicating combination of discharges involve same day events | (0) not a combined transfer or other same-day stay record, (1) combined transfer involving two discharges from different hospitals, (2) combined same-day stay involving two discharges at different hospitals, (3) combined same-day stay involving two discharges at the same hospital, (4) combined same-day stay involving three or more discharges at same or different hospitals |
| TOTCHG | Total charges (cleaned) |  |
| ZIPINC_QRTL | Median household income national quartile for patient ZIP Code | (1) $1 - $37,999; (2) $38,000 - $47,999; (3) $48,000 - $63,999; and (4) $64,000 or more. |
| APRDRG_Risk_Mortality | All Patient Refined DRG: Risk of Mortality Subclass | (0) No class specified, (1) Minor likelihood of dying, (2) Moderate likelihood of dying, (3) Major likelihood of dying, (4) Extreme likelihood of dying |
| APRDRG_Severity | All Patient Refined DRG: Severity of Illness Subclass | (0) No class specified, (1) Minor loss of function (includes cases with no comorbidity or complications), (2) Moderate loss of function, (3) Major loss of function, (4) Extreme loss of function |
| CM_AIDS | AHRQ comorbidity measure: Acquired immune deficiency syndrome | (1) comorbidity present, (0) comorbidity not present |
| CM_ALCOHOL | AHRQ comorbidity measure: Alcohol abuse | (1) comorbidity present, (0) comorbidity not present |
| CM_ANEMDEF | AHRQ comorbidity measure: Deficiency anemias | (1) comorbidity present, (0) comorbidity not present |
| CM_ARTH | AHRQ comorbidity measure: Rheumatoid arthritis/collagen vascular diseases | (1) comorbidity present, (0) comorbidity not present |
| CM_BLDLOSS | AHRQ comorbidity measure: Chronic blood loss anemia | (1) comorbidity present, (0) comorbidity not present |
| CM_CHF | AHRQ comorbidity measure: Congestive heart failure | (1) comorbidity present, (0) comorbidity not present |
| CM_CHRNLUNG | AHRQ comorbidity measure: Chronic pulmonary disease | (1) comorbidity present, (0) comorbidity not present |
| CM_COAG | AHRQ comorbidity measure: Coagulopathy | (1) comorbidity present, (0) comorbidity not present |
| CM_DEPRESS | AHRQ comorbidity measure: Depression | (1) comorbidity present, (0) comorbidity not present |
| CM_DM | AHRQ comorbidity measure: Diabetes, uncomplicated | (1) comorbidity present, (0) comorbidity not present |
| CM_DMCX | AHRQ comorbidity measure: Diabetes with chronic complications | (1) comorbidity present, (0) comorbidity not present |
| CM_DRUG | AHRQ comorbidity measure: Drug abuse | (1) comorbidity present, (0) comorbidity not present |
| CM_HTN_C | AHRQ comorbidity measure: Hypertension (combine uncomplicated and complicated) | (1) comorbidity present, (0) comorbidity not present |
| CM_HYPOTHY | AHRQ comorbidity measure: Hypothyroidism | (1) comorbidity present, (0) comorbidity not present |
| CM_LIVER | AHRQ comorbidity measure: Liver disease | (1) comorbidity present, (0) comorbidity not present |
| CM_LYMPH | AHRQ comorbidity measure: Lymphoma | (1) comorbidity present, (0) comorbidity not present |
| CM_LYTES | AHRQ comorbidity measure: Fluid and electrolyte disorders | (1) comorbidity present, (0) comorbidity not present |
| CM_METS | AHRQ comorbidity measure: Metastatic cancer | (1) comorbidity present, (0) comorbidity not present |
| CM_NEURO | AHRQ comorbidity measure: Other neurological disorders | (1) comorbidity present, (0) comorbidity not present |
| CM_OBESE | AHRQ comorbidity measure: Obesity | (1) comorbidity present, (0) comorbidity not present |
| CM_PARA | AHRQ comorbidity measure: Paralysis | (1) comorbidity present, (0) comorbidity not present |
| CM_PERIVASC | AHRQ comorbidity measure: Peripheral vascular disorders | (1) comorbidity present, (0) comorbidity not present |
| CM_PSYCH | AHRQ comorbidity measure: Psychoses | (1) comorbidity present, (0) comorbidity not present |
| CM_PULMCIRC | AHRQ comorbidity measure: Pulmonary circulation disorders | (1) comorbidity present, (0) comorbidity not present |
| CM_RENLFAIL | AHRQ comorbidity measure: Renal failure | (1) comorbidity present, (0) comorbidity not present |
| CM_TUMOR | AHRQ comorbidity measure: Solid tumor without metastasis | (1) comorbidity present, (0) comorbidity not present |
| CM_ULCER | AHRQ comorbidity measure: Peptic ulcer disease excluding bleeding | (1) comorbidity present, (0) comorbidity not present |
| CM_VALVE | AHRQ comorbidity measure: Valvular disease | (1) comorbidity present, (0) comorbidity not present |
| CM_WGHTLOSS | AHRQ comorbidity measure: Weight loss | (1) comorbidity present, (0) comorbidity not present |
