# Supplementary material for: Predicting all-cause 90-day hospital readmission for dental patients using machine learning methods
Source: BDJ Open. 2021 Jan 22;7:1. doi: 10.1038/s41405-021-00057-6 (PMC7822935; doi:10.1038/s41405-021-00057-6)
Supplement: Supplementary file 2 — Appendix B [file 41405_2021_57_MOESM2_ESM.docx]

**Appendix B: Top Primary dental diagnosis and procedure codes**

| **DX CODE** | **DESCRIPTION** | **PR CODE** | **DESCRIPTION** |
| --- | --- | --- | --- |
| 5225 | Periapical abscess without sinus | 2309 | Forceps; Extraction of other tooth |
| 5283 | Cellulitis and abscess of oral soft tissues | 2319 | Other surgical extraction of tooth |
| 5264 | Inflammatory conditions of jaw | 240 | Incision of gum or alveolar bone |
| 52100 | Dental caries, unspecified | 245 | Alveoloplasty |
| 5285 | Diseases of lips | 2301 | Forceps; Extraction of deciduous tooth |
| 52403 | Major anomalies of jaw size, maxillary hypoplasia | 9654 | Dental scaling, polishing, and debridement |
| 5224 | Acute apical periodontitis of pulpal origin | 2349 | Other dental restoration |
| 5227 | Periapical abscess with sinus | 2311 | Surgical; Removal of residual root |
| 52689 | Other specified diseases of the jaws | 247 | Application of orthodontic appliance |
| 5273 | Abscess of salivary gland | 232 | Restoration of tooth by filling |
