# Supplementary material for: Predicting all-cause 90-day hospital readmission for dental patients using machine learning methods
Source: BDJ Open. 2021 Jan 22;7:1. doi: 10.1038/s41405-021-00057-6 (PMC7822935; doi:10.1038/s41405-021-00057-6)
Supplement: Supplementary file 3 — Appendix C [file 41405_2021_57_MOESM3_ESM.docx]

**Appendix C: Summary by readmission group for 55 candidate variables.**

| **Variable** | **Readmission (N=1,746)** | **No Readmission (N=7,514)** | **P-value** |
| --- | --- | --- | --- |
| **LOS:** Mean (SD) | 12.8 (16.9) | 6.7 (13.9) | <0.001^t^ |
| **NRD_DaysToEvent:** Mean (SD) | 16,475.6 (3,138.2) | 16,476.0 (2,776.4) | 1.00^t^ |
| **Readmit:** No | 0 (0%) | 7514 (100%) | <0.001^c^ |
| Yes | 1,746 (100%) | 0 (0%) | - |
| **HOSP_BEDSIZE:** small | 62 (3.6%) | 377 (5%) | 0.001^c^ |
| medium | 274 (15.7%) | 1,358 (18.1%) | - |
| large | 1,410 (80.8%) | 5,779 (76.9%) | - |
| **H_CONTRL:** public | 375 (21.5%) | 1,504 (20%) | 0.020^c^ |
| voluntary | 1,237 (70.8%) | 5,281 (70.3%) | - |
| proprietary | 134 (7.7%) | 729 (9.7%) | - |
| **HOSP_URCAT4:** large metropolitan | 1,208 (69.2%) | 4,828 (64.3%) | <0.001^f^ |
| small metropolitan | 491 (28.1%) | 2,406 (32%) | - |
| micropolitan areas | 43 (2.5%) | 260 (3.5%) | - |
| not metropolitan or micropolitan | 4 (0.2%) | 20 (0.3%) | - |
| **HOSP_UR_TEACH:** metropolitan non-teaching | 337 (19.3%) | 1,746 (23.2%) | <0.001^c^ |
| metropolitan teaching | 1,362 (78%) | 5,488 (73%) | - |
| non-metropolitan | 47 (2.7%) | 280 (3.7%) | - |
| **AGE:** Mean (SD) | 49.8 (20.1) | 40.4 (20.6) | <0.001^t^ |
| **AWEEKEND:** admission on Monday-Friday | 1,353 (77.5%) | 5,915 (78.7%) | 0.26^c^ |
| admission on Saturday-Sunday | 393 (22.5%) | 1,599 (21.3%) | - |
| **DISPUNIFORM:** routine | 1,109 (63.5%) | 6,329 (84.2%) | <0.001^c^ |
| transfer to short term hospital | 19 (1.1%) | 34 (0.5%) | - |
| other transfers | 246 (14.1%) | 409 (5.4%) | - |
| home health care | 352 (20.2%) | 682 (9.1%) | - |
| against medical advice | 20 (1.1%) | 60 (0.8%) | - |
| **DQTR:** Jan-Mar | 560 (32.1%) | 2,347 (31.2%) | 0.61^c^ |
| Apr-Jun | 592 (33.9%) | 2,640 (35.1%) | - |
| Jul-Sep | 594 (34%) | 2,527 (33.6%) | - |
| **FEMALE:** male | 990 (56.7%) | 4,177 (55.6%) | 0.40^c^ |
| female | 756 (43.3%) | 3,337 (44.4%) | - |
| **HCUP_ED:** does not meet any HCUP ED criteria | 652 (37.3%) | 2,525 (33.6%) | 0.005^c^ |
| ED revenue code was on SID record | 735 (42.1%) | 3,201 (42.6%) | - |
| ED charge reported on SID record | 192 (11%) | 1,004 (13.4%) | - |
| other indication of ED services | 167 (9.6%) | 784 (10.4%) | - |
| **NCHRONIC:** Median (IQR) | 6.0 (3.0, 8.0) | 2.0 (1.0, 5.0) | <0.001^w^ |
| **NDX:** Median (IQR) | 14.0 (9.0, 18.0) | 7.0 (4.0, 12.0) | <0.001^w^ |
| **NECODE:** Median (IQR) | 0.0 (0.0, 1.0) | 0.0 (0.0, 0.0) | <0.001^w^ |
| **NPR:** Median (IQR) | 4.0 (2.0, 6.0) | 3.0 (2.0, 4.0) | <0.001^w^ |
| **ORPROC:** no major operating room procedure | 762 (43.6%) | 2,210 (29.4%) | <0.001^c^ |
| major operating room procedure | 984 (56.4%) | 5,304 (70.6%) | - |
| **PAY1:** Medicare | 645 (36.9%) | 1,454 (19.4%) | <0.001^c^ |
| Medicaid | 555 (31.8%) | 2,133 (28.4%) | - |
| private insurance | 310 (17.8%) | 1,954 (26%) | - |
| self-pay | 136 (7.8%) | 1,312 (17.5%) | - |
| no charge | 21 (1.2%) | 145 (1.9%) | - |
| other | 79 (4.5%) | 516 (6.9%) | - |
| **PL_NCHS:** Central counties ≥1 million population | 698 (40%) | 2,730 (36.3%) | 0.08^c^ |
| Fringe counties ≥1 million population | 389 (22.3%) | 1,699 (22.6%) | - |
| Counties of 250,000-999,999 population | 282 (16.2%) | 1,363 (18.1%) | - |
| Counties of 50,000-249,999 population | 143 (8.2%) | 679 (9%) | - |
| Micropolitan counties | 133 (7.6%) | 595 (7.9%) | - |
| Not metropolitan or micropolitan counties | 101 (5.8%) | 448 (6%) | - |
| **REHABTRANSFER:** No | 1,719 (98.5%) | 7,455 (99.2%) | 0.003^c^ |
| Yes | 27 (1.5%) | 59 (0.8%) | - |
| **RESIDENT:** No | 85 (4.9%) | 383 (5.1%) | 0.69^c^ |
| Yes | 1,661 (95.1%) | 7,131 (94.9%) | - |
| **SAMEDAYEVENT:** not a combined transfer | 1,603 (91.8%) | 7,240 (96.4%) | <0.001^c^ |
| combined transfer:two discharges from different hospitals | 70 (4%) | 113 (1.5%) | - |
| combined same-day stay:two discharges at different hospitals | 32 (1.8%) | 60 (0.8%) | - |
| combined same-day stay:two discharges at the same hospital | 27 (1.5%) | 69 (0.9%) | - |
| combined same-day stay:three or more discharges at same or different hospitals | 14 (0.8%) | 32 (0.4%) | - |
| **TOTCHG:** Mean (SD) | 124,005.3 (216,769.3) | 64,857.2 (115,996.4) | <0.001^t^ |
| **ZIPINC_QRTL:** $1 - $37,999 | 581 (33.3%) | 2,543 (33.8%) | 0.60^c^ |
| $38,000 - $47,999 | 436 (25%) | 1,955 (26%) | - |
| $48,000 - $63,999 | 410 (23.5%) | 1,669 (22.2%) | - |
| $64,000 or more | 319 (18.3%) | 1,347 (17.9%) | - |
| **APRDRG_Risk_Mortality:** Minor likelihood of dying | 701 (40.1%) | 5,424 (72.2%) | <0.001^c^ |
| Moderate likelihood of dying | 534 (30.6%) | 1,187 (15.8%) | - |
| Major likelihood of dying | 373 (21.4%) | 634 (8.4%) | - |
| Extreme likelihood of dying | 138 (7.9%) | 269 (3.6%) | - |
| **APRDRG_Severity:** Minor loss of function | 270 (15.5%) | 3,356 (44.7%) | <0.001^c^ |
| Moderate loss of function | 566 (32.4%) | 2,560 (34.1%) | - |
| Major loss of function | 633 (36.3%) | 1,164 (15.5%) | - |
| Extreme loss of function | 277 (15.9%) | 434 (5.8%) | - |
| **CM_AIDS:** comorbidity not present | 1,729 (99%) | 7,489 (99.7%) | <0.001^c^ |
| comorbidity present | 17 (1%) | 25 (0.3%) | - |
| **CM_ALCOHOL:** comorbidity not present | 1,576 (90.3%) | 7,041 (93.7%) | <0.001^c^ |
| comorbidity present | 170 (9.7%) | 473 (6.3%) | - |
| **CM_ANEMDEF:** comorbidity not present | 1,350 (77.3%) | 6,712 (89.3%) | <0.001^c^ |
| comorbidity present | 396 (22.7%) | 802 (10.7%) | - |
| **CM_ARTH:** comorbidity not present | 1,695 (97.1%) | 7,374 (98.1%) | 0.005^c^ |
| comorbidity present | 51 (2.9%) | 140 (1.9%) | - |
| **CM_BLDLOSS:** comorbidity not present | 1,717 (98.3%) | 7,471 (99.4%) | <0.001^c^ |
| comorbidity present | 29 (1.7%) | 43 (0.6%) | - |
| **CM_CHF:** comorbidity not present | 1,582 (90.6%) | 7,255 (96.6%) | <0.001^c^ |
| comorbidity present | 164 (9.4%) | 259 (3.4%) | - |
| **CM_CHRNLUNG:** comorbidity not present | 1,358 (77.8%) | 6,540 (87%) | <0.001^c^ |
| comorbidity present | 388 (22.2%) | 974 (13%) | - |
| **CM_COAG:** comorbidity not present | 1,546 (88.5%) | 7,202 (95.8%) | <0.001^c^ |
| comorbidity present | 200 (11.5%) | 312 (4.2%) | - |
| **CM_DEPRESS:** comorbidity not present | 1,536 (88%) | 6,947 (92.5%) | <0.001^c^ |
| comorbidity present | 210 (12%) | 567 (7.5%) | - |
| **CM_DM:** comorbidity not present | 1,406 (80.5%) | 6,585 (87.6%) | <0.001^c^ |
| comorbidity present | 340 (19.5%) | 929 (12.4%) | - |
| **CM_DMCX:** comorbidity not present | 1,630 (93.4%) | 7,348 (97.8%) | <0.001^c^ |
| comorbidity present | 116 (6.6%) | 166 (2.2%) | - |
| **CM_DRUG:** comorbidity not present | 1,554 (89%) | 6,981 (92.9%) | <0.001^c^ |
| comorbidity present | 192 (11%) | 533 (7.1%) | - |
| **CM_HTN_C:** comorbidity not present | 944 (54.1%) | 5,263 (70%) | <0.001^c^ |
| comorbidity present | 802 (45.9%) | 2,251 (30%) | - |
| **CM_HYPOTHY:** comorbidity not present | 1,632 (93.5%) | 7,088 (94.3%) | 0.17^c^ |
| comorbidity present | 114 (6.5%) | 426 (5.7%) | - |
| **CM_LIVER:** comorbidity not present | 1,630 (93.4%) | 7,320 (97.4%) | <0.001^c^ |
| comorbidity present | 116 (6.6%) | 194 (2.6%) | - |
| **CM_LYMPH:** comorbidity not present | 1,716 (98.3%) | 7,470 (99.4%) | <0.001^c^ |
| comorbidity present | 30 (1.7%) | 44 (0.6%) | - |
| **CM_LYTES:** comorbidity not present | 1,219 (69.8%) | 6,202 (82.5%) | <0.001^c^ |
| comorbidity present | 527 (30.2%) | 1,312 (17.5%) | - |
| **CM_METS:** comorbidity not present | 1,683 (96.4%) | 7,409 (98.6%) | <0.001^c^ |
| comorbidity present | 63 (3.6%) | 105 (1.4%) | - |
| **CM_NEURO:** comorbidity not present | 1,594 (91.3%) | 7,090 (94.4%) | <0.001^c^ |
| comorbidity present | 152 (8.7%) | 424 (5.6%) | - |
| **CM_OBESE:** comorbidity not present | 1,528 (87.5%) | 6,796 (90.4%) | <0.001^c^ |
| comorbidity present | 218 (12.5%) | 718 (9.6%) | - |
| **CM_PARA:** comorbidity not present | 1,686 (96.6%) | 7,354 (97.9%) | 0.001^c^ |
| comorbidity present | 60 (3.4%) | 160 (2.1%) | - |
| **CM_PERIVASC:** comorbidity not present | 1,625 (93.1%) | 7,352 (97.8%) | <0.001^c^ |
| comorbidity present | 121 (6.9%) | 162 (2.2%) | - |
| **CM_PSYCH:** comorbidity not present | 1,608 (92.1%) | 7,152 (95.2%) | <0.001^c^ |
| comorbidity present | 138 (7.9%) | 362 (4.8%) | - |
| **CM_PULMCIRC:** comorbidity not present | 1,680 (96.2%) | 7,420 (98.7%) | <0.001^c^ |
| comorbidity present | 66 (3.8%) | 94 (1.3%) | - |
| **CM_RENLFAIL:** comorbidity not present | 1,496 (85.7%) | 7,187 (95.6%) | <0.001^c^ |
| comorbidity present | 250 (14.3%) | 327 (4.4%) | - |
| **CM_TUMOR:** comorbidity not present | 1,689 (96.7%) | 7,430 (98.9%) | <0.001^c^ |
| comorbidity present | 57 (3.3%) | 84 (1.1%) | - |
| **CM_ULCER:** comorbidity not present | 1,745 (99.9%) | 7,514 (100%) | 0.19^f^ |
| comorbidity present | 1 (0.1%) | 0 (0%) | - |
| **CM_VALVE:** comorbidity not present | 1,636 (93.7%) | 7,283 (96.9%) | <0.001^c^ |
| comorbidity present | 110 (6.3%) | 231 (3.1%) | - |
| **CM_WGHTLOSS:** comorbidity not present | 1,569 (89.9%) | 7,211 (96%) | <0.001^c^ |
| comorbidity present | 177 (10.1%) | 303 (4%) | - |

^t^ T-test, ^c^ Chi-squared test, ^f^ Fisher's exact test, ^w^ Wilcoxon rank sum test.
